# Interobserver Agreement in Histopathological Subtyping of Malignant Pleural Mesotheliomas

**DOI:** 10.5146/tjpath.2020.01498

**Published:** 2021-01-15

**Authors:** Mona Mlika, Faouzi Mezni

**Affiliations:** Department of Pathology, Abderrahman Mami Hospital, University of Medicine of Tunis, University of Tunis El Manar, Research Laboratory: LR18SP06, Tunis, Tunisia

**Keywords:** Interobserver agreement, Malignant pleural mesothelioma, Mesothelioma subtypes

## Abstract

***Objective:*** Many recent studies are pointing out the heterogeneity between pathologists in the classification of malignant pleural mesotheliomas. Besides, they reported the prognostic impact of classifying epithelioid mesotheliomas according to the predominant architectural features and the nuclear grade. The authors assessed the interobserver and the intraobserver agreement of subtyping mesotheliomas between 2 pathologists used to thoracic pathology.

***Material and Method:*** The observers reviewed all the slides of the malignant pleural mesotheliomas diagnosed during the period ranging from 2004 to 2017. The Cohen Kappa was performed in order to evaluate the agreement between both observers into classifying mesotheliomas, subtyping and grading epithelioid mesotheliomas. Two rounds of examination were planned with a delay period of one month. After the first round, the reviewers discussed the different difficulties and challenges they faced. All the statistic tests were performed using the SPSS software version 12.0.

***Results:*** After the first round, a fair agreement between both observers was reported. After the second round, an improvement of the concordance rate with a good agreement in subtyping epithelioid mesotheliomas was noticed. Concerning the grading of mesotheliomas, the interobserver agreement was poor even after the second round examination. The intraobserver reproducibility of epithelioid mesothelioma subtyping was fair or moderate for both reviewers. The intraobserver agreement was poor concerning the grading of epithelioid mesothelioma.

***Conclusion:*** Integrating subtyping and grading of epithelioid mesotheliomas into a new classification necessitates an important training of the pathologists. The architectural features’ definitions have to be clarified in order to avoid using own subjective opinions and habits by pathologists.

## INTRODUCTION

Malignant pleural mesothelioma is a rare tumour with a poor prognosis. The management of these tumours is based on a multidisciplinary approach associating surgical resection, radiation therapy and chemotherapy. The most relevant prognostic factors consist in the stage and the histologic subtype. The microscopic diagnosis is made in accordance with the 2015 World Health Organization Classification (1). This classification made huge modifications in lung cancer classification concerning the subtyping of adenocarcinomas according to the predominant architecture. On the other hand, it put emphasis on the heterogeneous architecture of epithelioid pleural mesotheliomas. In fact, pleural mesotheliomas are classified into diffuse or localized tumours based on their distribution and into epithelioid, sarcomatoid or biphasic tumours according to their microscopic features. These tumours present different prognoses. The best prognosis is attributed to epithelioid tumours and the worse one to sarcomatoid tumours. As it was noticed in lung primary adenocarcinomas, the group of epithelioid mesotheliomas seemed to be heterogeneous in terms of prognoses, molecular features and microscopic aspects. Many different architectural subtypes have been identified into the WHO classification and reported by the international mesothelioma interest group (2–5). These subtypes consisted of solid, trabecular, acinar, tubulo-papillary, micropapillary, adenomatoid, deciduoid and transitional. Every subtype is characterized by specific diagnostic features, which were slightly described in the WHO classification (1). Many authors reported the different prognostic impact of these subtypes pointing out the poor prognosis of transitional epithelioid mesotheliomas that was reported to be similar to sarcomatoid mesotheliomas (6). In addition to these different subtypes, many authors reported the prognostic impact of grading epithelioid mesotheliomas. The grading system takes into account nuclear grade, mitoses and necrosis (7). It has been reported to be a relevant prognostic feature by many authors. Recently, the international mesothelioma interest group stipulated the necessity of reporting the subtype of epithelioid mesotheliomas in addition to the grade (3,5). This fact made us wonder about the reproducibility of these features.

The authors aimed to assess the inter-observer and intra-observer reproducibility of classifying pleural mesotheliomas and subtyping epithelioid mesotheliomas according to the predominant architecture and the grade.

## MATERIAL and METHODS

### Study Design

The authors reported a descriptive, retrospective and transversal study including pleural mesotheliomas diagnosed in a single institution. The authors reviewed all the slides of the malignant pleural mesotheliomas diagnosed during the period ranging from 2004 and 2017. They reviewed hematoxylin eosin stain slides and silanized slides used for immunohistochemistry.

### Inclusion Criteria

All the malignant pleural mesotheliomas diagnosed during the period of the study were retrieved from the archives of the Department of Pathology.

### Exclusion Criteria

The specimen for which no paraffin blocks were available, were excluded.

### Non-Inclusion Criteria

Other pleural tumours were excluded from this study.

### Reviewers’ Characteristics

All the slides were reviewed by 2 pathologists who were used to thoracic pathology. The first pathologist had 25 years of experience and the second pathologist had 10 years of experience in thoracic pathology.

### Slides Reviewing

The authors were given a sheet with different items to record including the number of slides reviewed, the number of samples, the mesothelioma subtypes including epithelioid, sarcomatoid, biphasic and the architecture-based subtyping in case of epithelioid mesothelioma including trabecular, solid, micropapillary, tubulo-papillary, acinar, adenomatoid, transitional, deciduoid or special variant including pleomorphic cells or signet ring cells. The grade was also recorded in epithelioid mesotheliomas. Two rounds of evaluation, with an interval of one month, were performed. After the first round, the two reviewers met for a clarification session to present the different difficulties and challenges they faced.

### Subtyping Criteria

**Figure 1:**
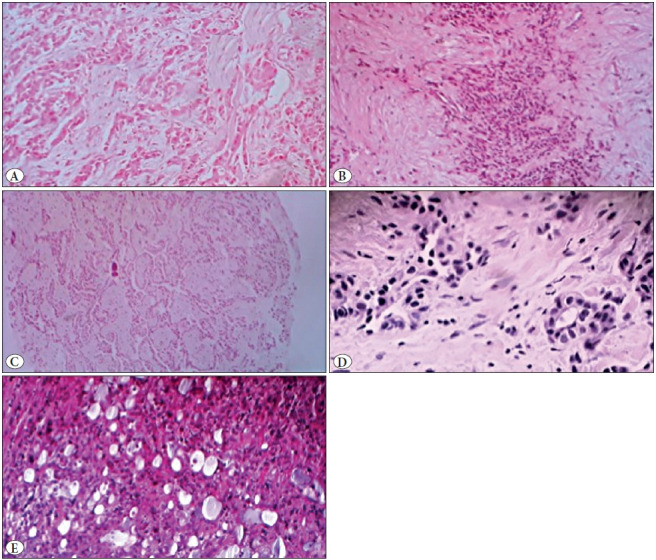
**A)** The trabecular subtype with small cells arranged into thin cords or single files (H&E; x400). **B)** The solid subtype with nests of tumour cells (H&E; x200). **C)** The papillary subtype with papillary structures with a fibrovascular core. **D)** Acinar structures with glandular structures (H&E; x200). **E)** The adenomatoid structures with pseudoglandular structures (H&E; x200).

Subtyping of pleural mesothelioma was made according to the WHO classification (1). Epithelioid mesothelioma was defined as a tumour made of polygonal or ovoid cells. Sarcomatoid mesothelioma was characterized by elongated and tapered mesothelial cells with various degrees of atypia and mitoses. Biphasic tumours were defined by the association of an epithelioid component to a sarcomatoid one with a minimum proportion of 10 % for each component. Concerning the epithelioid mesotheliomas, the trabecular subtype consisted in small cells arranged into thin cords or single files (Figure 1). Nests of tumour cells defined the solid subtype (Figure 1). Papillary subtype was characterized by papillary structures with a fibrovascular core (Figure 1). Micropapillary structures were digitiform structures without a fibrovascular core. Acinar structures were glandular structures (Figure 1). Adenomatoid structures were characterized by pseudoglandular structures (Figure 1). Deciduoid subtype was composed by large cells with atypical nuclei, abundant cytoplasm with pronounced eosinophilia, and glassy cytoplasm mimicking deciduoid cells. The description of the transitional subtype was not clear in the WHO classification, and that is why the authors adopted the definition of Galateau Sallé, et al. Transitional subtype was defined as sheets of plump cells starting to lose their epithelioid morphology but not overtly spindle shaped and lacking frank sarcomatous features (6).

**Figure 2:**
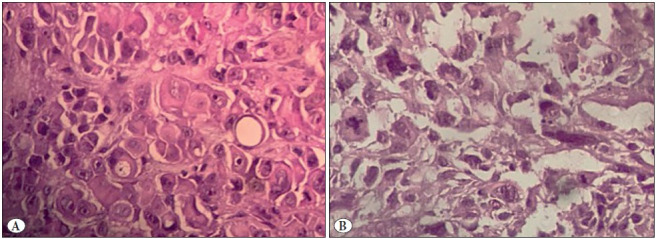
**A)** Epithelioid mesothelioma characterized by a low nuclear grade with regular nuclei and rare mitoses (H&E; x400).** B)** Epithelioid mesothelioma characterized by a high-grade nuclear grade with atypical nuclei and numerous mitoses (H&E; x400).

Grading was performed according to the IASLC proposition criteria and was based on the nuclear grading, mitotic index and necrosis (3). A two-tier system was established with low-grade tumours consisting of grade I or II tumours without necrosis and high-grade tumours consisting of grade III tumours and grade II tumours with necrosis. Nuclear grade was scored 1, 2 or 3 for respectively mild, moderate or severe nuclear atypia. Mitotic count was scored 1, 2 or 3 for tumours with respectively less than 1 mitosis per 2 mm2, 2 to 4 mitoses per 2 mm2 and more than 5 mitoses per 2 mm2. A sum of 2 or 3 was considered as a grade I, a sum of 4 or 5 was considered as a grade II, and a sum of 6 was consistent with grade III tumour. The reviewers had no limit of time to review the cases (Figure 2).

### Statistical Analysis

The Cohen Kappa was performed in order to evaluate the agreement between both pathologists into classifying mesotheliomas, subtyping epithelioid mesotheliomas and reporting the grade in epithelioid mesothelioma.

Two rounds of examination were planned with a delay period of one month. After the first round, the reviewers discussed the different difficulties and challenges they faced. Interobserver agreement and intraobserver agreement were performed. The strength of agreement was considered excellent for kappa >0.8, good for 0.61<Kappa<0.80, moderate for 0.41<Kappa<0.6, fair for 0.21<Kappa<0.4, poor for 0.00<Kappa<0.2 and very poor for Kappa<0.00. The proportion of concordant cases was assessed. All the statistic tests were performed using the SPSS software version 12.0.

### Ethics Considerations

All the blocks included were anonymized and no information concerning the patients was used in this study. A reference number was attributed to each case.

## RESULTS

The diagnosis of malignant mesothelioma was made on needle biopsies in 16 cases, thoracoscopic biopsy in 32 cases, and surgical specimens in 2 cases. The latter consisted of a bullectomy and an extra pleural pneumonectomy. Total concordance between pathologists was observed in 10/16 needle biopsies, 21/32 thoracoscopic biopsies, and 1/2 surgical specimens. Both pathologists reviewed the same number of slides.

### Interobserver Reproducibility Concerning the Subtyping of Mesotheliomas

This study included 50 malignant pleural mesotheliomas. Both pathologists reviewed all cases with a mean of 7 blocks (slides)/ case (min 1 block, max 21 blocks). The different cases consisted of epithelioid mesotheliomas in 44 cases (88%), sarcomatoid mesothelioma in 4 cases (8%), and biphasic mesothelioma in 2 cases (4%).

**Figure 3:**
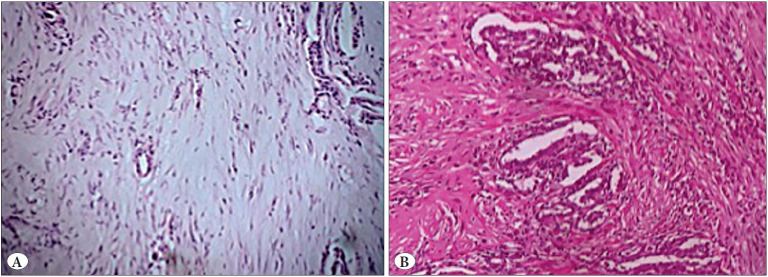
**A)** Case 15 was considered as a trabecular subtype by observer 1 and acinar subtype by observer 2 (H&E; x200). **B)** Case 35 was considered as biphasic mesothelioma by observer 2 and tubulopapillary mesothelioma by observer 1 (H&E; x200).

The concordance rate accounted for 32% (16/50) when taking into account the subtyping and the grading. It reached 44% (22/50) when taking into account only the subtyping of mesotheliomas without the grading. The pathologists were confused when differentiating solid from deciduoid subtypes (4 cases), papillary from trabecular subtypes (4 cases), acinar from trabecular subtypes (4 cases), signet-ring cell from solid subtypes (2 cases), trabecular from micropapillary subtype (2 cases), solid from trabecular subtype (2 cases), solid from acinar subtype (2 cases), sarcomatoid from pleomorphic (2 cases), sarcomatoid from trabecular subtypes (2 cases), and biphasic from epithelioid subtypes (4 cases) (Figure 3). The highest concordance rates were observed in the tubulo-papillary, trabecular, solid, biphasic, and sarcomatoid subtypes with ratios reaching respectively 8/12, 14/18, 6/6, 2/2 and 4/6. The deciduoid and the acinar subtypes presented the lowest concordance rates, reaching 0/2 and 2/4 respectively. The first reviewer recognized 8 trabecular mesotheliomas, 6 acinar, 6 solid, 14 tubulo-papillary, 2 micropapillary, and 8 special variants consisting of 2 pleomorphic cases, 2 signet ring cells cases, 2 deciduoid cases, and 6 sarcomatoid mesotheliomas. The second reviewer identified 14 trabecular mesotheliomas that were judged by the first reviewer as trabecular in 4 cases, acinar in 4 cases, tubulo-papillary in 2 cases, micropapillary in 2 cases, and sarcomatoid in 2 cases. The second reviewer recognized 16 solid mesotheliomas classified by the first reviewer as trabecular in 2 cases, acinar in 2 cases, and solid in 6 cases. The second reviewer identified also 6 biphasic mesotheliomas classified as trabecular in 2 cases, papillary in 2 cases, and a special variant in 6 cases by the first reviewer. The Cohen Kappa reached the value of 0.34 corresponding to a fair agreement.

After the second session, the inter-observer agreement was good with a weighted Kappa value of 0.62. The highest concordance rates were observed for solid, acinar, and sarcomatoid subtypes reaching 100%. The concordance rate accounted for 50% for papillary subtype.

### Interobserver Reproducibility Concerning Nuclear Grading

Concerning the nuclear grading of the epithelioid mesotheliomas, the first reviewer considered 16 high-grade and 28 low-grade tumours. The second reviewer considered 22 low-grade and 22 high-grade tumours. Among the 32 high-grade tumours recorded by the second reviewer, 16 cases were also considered as high-grade by the first reviewer. Among the 12 low-grade tumours recorded by the second reviewer, 10 cases were also recorded as low-grade by the first one. The concordance between the judges accounted for 59% (26/44). The agreement between the reviewers was fair (Cohen Kappa=0.28). After the second round, the agreement of nuclear grade was poor with a weighted Kappa accounting for 0.

### Intraobserver Reproducibility Concerning the Subtyping of Mesotheliomas and the Grading of Epithelioid Mesothelioma

Between both sessions of examination, the intraobserver agreement of mesothelioma subtyping was fair (Kap-pa=0.27) and moderate (Kappa=0.44) for the first and the second reviewer respectively. For the first reviewer, the worse concordance rates were recorded for solid, trabecular, acinar, the special variants, and micro-papillary subtypes. For the second reviewer, the worse concordance rates were recorded for the trabecular and biphasic subtypes.

Concerning the grading, the intraobserver agreement was very poor (Kappa=0) and poor (Kappa=0.2) for reviewer 1 and 2 respectively.

The different values of the weighted kappa are represented in Table 1.

**Table 1:** The distribution of kappa scores for subtyping mesotheliomas and assessing nuclear grade.

	**Interobserver reproducibility**				**Intraobserver reproducibility**			
	**Subtyping mesothelioma** **First round**	**Subtyping mesothelioma** **Second round**	**Nuclear grade** **First round**	**Nuclear grade** **Second round**	**Subtyping mesothelioma**		**Nuclear grade**	
					**Obs1**	**Obs 2**	**Obs 1**	**Obs 2**
Weighted Kappa coefficient	0.34	0.62	0.28	0	0.27	0.44	0	0.2

Strength of agreement according to Kappa value: excellent: K>0.8, good: 0.61<K<0.8, moderate 0.41<K<0.6, fair 0.21<K<0.4, poor: 0.00<K<0.20, very poor: K<0.0

## DISCUSSION

In this retrospective and descriptive study, the authors assessed the reproducibility of subtyping the pleural mesotheliomas after two-round sessions. They included the subtype and grade of epithelioid mesotheliomas. The second round was performed after a clarification session between the 2 reviewers who were used to practicing Thoracic Pathology. During the first round, they reported a fair agreement between both observers concerning mesothelioma subtyping and the grading. After the second round, they noticed an improvement of the concordance rate with a good agreement in subtyping epithelioid mesotheliomas. Concerning the grading, the interobserver agreement was poor even after the second round examination. The intraobserver agreement of epithelioid mesothelioma subtyping was fair or moderate for both reviewers. The intra-observer agreement was poor concerning the grading. This kind of study seems necessary in order to assess the validity of new criteria that can be integrated in these tumours’ classification. The 2015 World Health Organization Classification of lung cancer resulted from numerous studies that reported the heterogeneity of the adenocarcinomas and the reproducibility of a classification based on the major architectural subtypes (1). In this classification, the heterogeneity of the epithelioid mesotheliomas was pointed out without a real recommendation to subtype these tumours according to the predominant architectural feature. The prognostic impact of subtyping epithelioid mesotheliomas was reported by many authors (2,5–8). Rosen L, et al. reported a better prognosis of mesotheliomas with trabecular or tubulo-papillary patterns in comparison to those with other patterns (7). In a study including 108 pleural mesotheliomas, Brcic L, et al. reported a good agreement with a Kappa coefficient reaching 0.72 (9). In 2018, the same authors assessed the interobserver and intraobserver agreement between the main types of mesotheliomas and the subtypes of epithelioid mesothelioma in a study about 200 patients. In opposition to their first manuscript, they reported a fair interobserver agreement, which was substantially improved after a clarification session between the observers. In this study, the clarification between both reviewers induced an improved agreement between pathologists. The clarification had no effect on the reproducibility of the grading of epithelioid mesotheliomas. In this study, the clarification between both observers made the first observer realize the accurate definition of the special variants including deciduoid or signet ring cell mesothelioma. Brcic L, et al. noticed the highest agreement for sarcomatoid and epithelioid mesotheliomas and the lowest agreement for biphasic ones (9). In this study, the most reproducible subtypes consisted in solid, tubulo-papillary, and sarcomatoid subtypes. Difficulties in classifying biphasic tumours may be explained by the cut-off of 10%, which may be difficult to be assessed unanimously or the difficulty of highlighting the sarcomatoid component, which can be confused with active fibroblasts. The ASCO guideline for the treatment and diagnosis of malignant mesothelioma made a recommendation to quantify epithelioid versus sarcomatoid components in surgical, thoracoscopic or open pleural biopsies (4). In opposition to these guidelines, the reproducibility of classifying biphasic mesotheliomas between MESOPATH pathologists and the International Mesothelioma Panel pathologists was reported to be moderate with a weighted Kappa value of 0.45 (10). Brcic L attributed the better reproducibility of tubulo-papillary, pleomorphic and trabecular patterns to the “striking” character of these patterns that can be easily identified and may be over-estimated by the pathologists (9). The most challenging pattern reported by the authors and which was not reported a few years before was the acinar pattern. This fact was also noticed in this study and can be explained by the difficulties to differentiate trabecular pattern from acinar one when dealing with slit-like spaces. Acinar and adenomatoid patterns may be also difficult to distinguish because of the definition of glands and of differentiating them from microcysts. After the second round, Brcic et al. reported the highest improvements in micropapillary, deciduoid and solid patterns with the same concern for the acinar pattern (11). The transitional subtype was not reported in this study. It has been clearly defined by Galateau Salle, et al. (6). This subtype was reported to be hardly distinguished from sarcomatoid subtype. Dacic S, et al. reported a fair interobserver agreement for diagnosing transitional subtype and an interobserver agreement dependant on the percentage of specific foci when dealing with sarcomatoid features. This agreement was excellent when the proportion of sarcomatoid features accounted for more than 75% (12). Some authors reported that the sample size may be a limiting factor because they noticed a low agreement between observers when dealing with needle biopsies (11). These results were in contradiction with those of Chirieac LR et al. who reported a high concordance of the diagnosis made on needle biopsies and surgical biopsies in a study on 759 cases (13). They put emphasis on the high accuracy of biopsies in sarcomatoid subtypes in comparison to epithelioid subtypes. Besides, they reported that the accuracy of histologic classification increases with the number of tissue blocks examined with a 100% concordance when more than 9 biopsies were included. Another limitation reported by Crzs et al. was the pathologists’ expertise. In fact, they reported moderate intraobserver agreement with the lowest value attributed to the least experienced pathologist. This fact was not reflected by this study’s results because the highest intraobserver variability was attributed to the most experienced pathologist. This study puts emphasis on the difficulties of subtyping the epithelioid mesotheliomas. These difficulties are added to the challenges described when differentiating the mesotheliomas from the multiple mimickers including lung or breast carcinomas (14). In spite of all these difficulties, pathologists have to adopt the subtyping of epithelioid mesotheliomas because morphologic features reflect molecular pathways. Blum Y, et al. reported that mesotheliomas may be decomposed as a combination of epithelioid-like and sarcomatoid-like components that reflect different oncogenic pathways and whose proportions are highly related to the prognosis (15).

In conclusion, this study puts emphasis on the difficulty of subtyping epithelioid mesotheliomas according to their architecture features and grade. Training and more accurate details in the definition of the different features are needed in order to integrate these characteristics in the classification of malignant mesotheliomas and to make them relevant in predicting the prognosis of mesotheliomas.

## CONFLICT of INTEREST

The authors declare no conflict of interest.

## FUNDING

The authors declared that this study has received no financial support.
